# Metanephric Adenofibroma in a young adult

**DOI:** 10.1590/S1677-5538.IBJU.2015.0300

**Published:** 2017

**Authors:** Feiya Yang, Canfeng Zhang, Mingshuai Wang, Xiquan Tian, Wenlong Wang, Nianzeng Xing

**Affiliations:** 1 Department of Urology, Beijing Chaoyang Hospital, Capital Medical University, Beijing, P.R. China;; 2 Department of Orthopedics, Beijing Chaoyang Hospital, Capital Medical University, Beijing, P.R. China

## CASE

**Figure 1:**
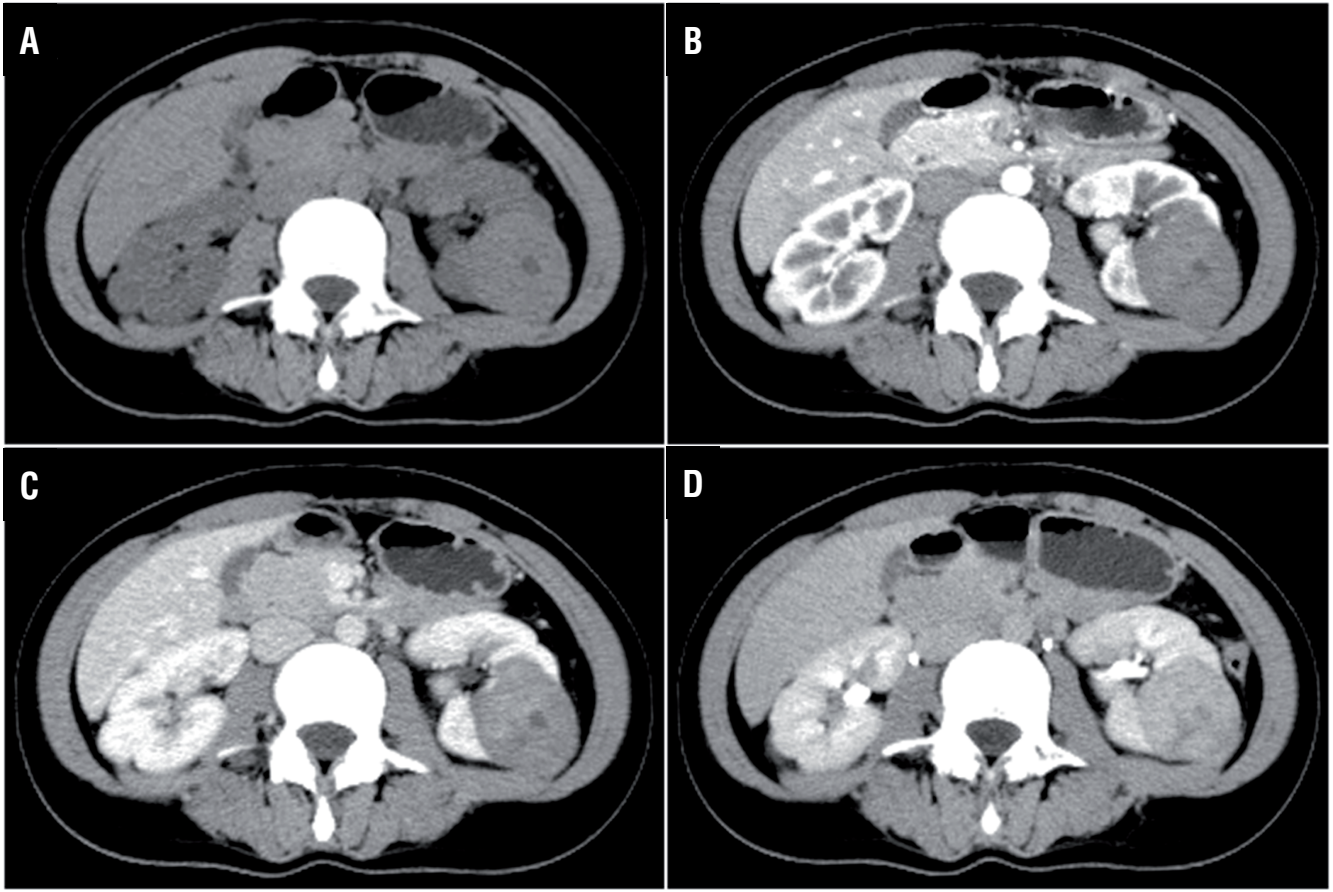
Enhanced computed tomography scan (CT) demonstrated a 4.5×4.0×4.5cm neoplasm in the middle and dorsal part of left kidney (CT unit: 45). It had a clear boundary and part of the neoplasm extruded the renal contour. Multiple patchy low density area could be seen in the neoplasm (CT unit: 10). The neoplasm was lightly enhanced in the arterial phase (CT unit: 57.2), and it was continuously enhanced in the venous (CT unit: 68.9) and excretory phase (CT unit: 88.8). The low density area did not show any enhancement.

**Figure 2:**
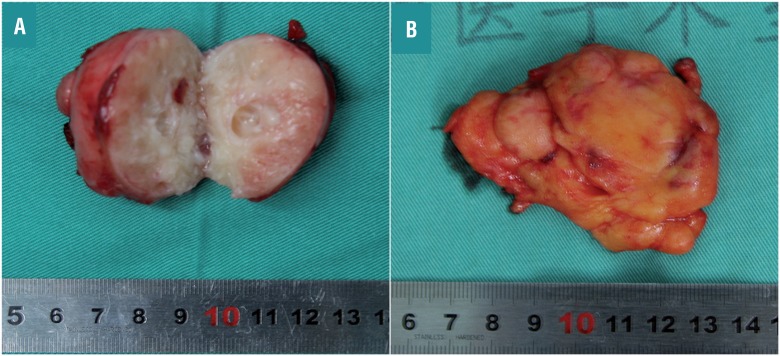
Gross pathologic features of the resected specimen. The cut section indicated a red-white cystic solid tumor mass measuring 5×4cm (A). The lesion was completely resected and accompanied with some fat covering the outer surface (B).

**Figure 3:**
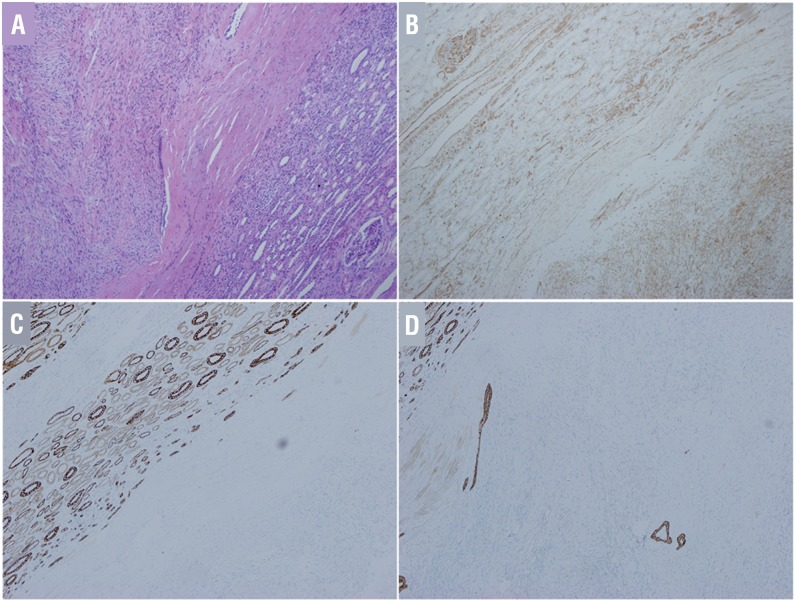
Postoperative pathological examination verified metanephric adenofibroma (×100). (A) Spindle cells proliferate and the cell size is consistent with a few acini and tubular structures scattering among them and some are cystic expansion. The lined epithelial cells did not show atypia. Immunohistochemical results show that vimentin is positive for tumor (B) and CK is positive for small tube and negative for tumor (C, D).

A 29-year-old female patient was admitted after a computed tomography (CT) scan showing a neoplasm measuring 4.5×4.0×4.5cm located in the middle and dorsal part of the left kidney (Figure-1). In the CT plain scan phase, the neoplasm had almost equal density with the normal kidney, and the CT unit was 45. It had a clear boundary, and part of the neoplasm extruded the renal contour. A multiple patchy low density area could be seen in the neoplasm that had a CT unit of 10. The renal sinus and calyx were slightly squeezed (Figure-1A). In the arterial phase, the neoplasm was slightly homogenously enhanced, and the CT unit was 57.2. The boundary was clear, and no obvious enhancement was manifested in the low density area. No other enhanced or abnormal low density foci were observed in the remaining renal parenchyma (Figure-1B). In the venous phase, the neoplasm was continuously enhanced and the CT unit was 68.9 (Figure-1C). In the excretory phase, the neoplasm was persistly enhanced, and the CT unit was 88.8. The low density area did not show obvious enhancement, and there was no change in compression or obvious destruction in the renal sinus and calyx. The perirenal fatty gap was clear (Figure-1D). All of the manifestations were different from that of renal malignant tumors. A laparoscopic partial nephrectomy under general anesthesia was performed to completely resect the lesion. Gross examination indicated a red-white cystic solid tumor mass measuring 5×4cm (Figure-2). There was liquefaction and necrosis in the center of this neoplasm. Postoperative histopathologic examination verified that it was metanephric adenofibroma (Figure-3). The patient was discharged on postoperative day 5, and no recurrence or metastasis was observed during the 8 months of postoperative follow-up.

**A** = CT plain scan. B = The arterial phase. **C** = The venous phase. **D** = The excretory phase.

MAF (metanephric adenofibroma), initially reported in 1992 (1), is a rare metanephric renal tumor that occurs primarily in children and young adults (2). Histopathologic examination reveals MAF to be a benign tumor that is composed of varying proportions of epithelial-stromal elements and immunohistochemistry can differentiate it from Wilms tumor and papillary renal cell carcinoma mainly using CK, vimentin, CD34 and cytokeratin 7 (3). Galluzzo ML (4) reported a case with simultaneous MAF, Wilms tumor and clear cell carcinoma, indicating a relationship between these tumors.

Herein, we describe the CT manifestations of MAF to help make the accurate preoperative diagnosis of MAF, the diagnostic methods of which have not been sufficiently reported. Currently, its confirmed diagnosis mainly depends on postoperative pathology. As for the best treatment modality, surgery is preferred, among which laparoscopic partial nephrectomy is ideal when conditions permit. Although no postoperative recurrence is reported, long-term follow-up is still needed. More clinical cases are required to facilitate formulations of standard treatment and follow-up for MAF.
